# The Impact of Frailty Indices on Predicting Complications and Functional Recovery in Proximal Humerus Fractures: A Comparative Study

**DOI:** 10.3390/medicina61071169

**Published:** 2025-06-27

**Authors:** Ekrem Özdemir, Oya Olcay Özdeş, Fatih Emre Topsakal, Nasuhi Altay, Esra Demirel

**Affiliations:** 1 Department of Orthopedics and Traumatology, Erzurum City Hospital, 25240 Erzurum, Türkiye; drfatihtopsakal@hotmail.com (F.E.T.); onasuhialtay@hotmail.com (N.A.); esrademirel82@gmail.com (E.D.); 2 Department of Anesthesiology and Reanimation, Battalgazi State Hospital, 44320 Malatya, Türkiye; oyayiilmaz@hotmail.com

**Keywords:** proximal humerus fracture, frailty indices, modified frailty index, functional outcome, prognosis, geriatric trauma, orthopedic complications

## Abstract

*Background and Objectives:* This retrospective cohort study aimed to evaluate the predictive validity of four frailty indices—Modified Frailty Index-5 (mFI-5), Edmonton Frail Scale (EFS), Clinical Frailty Scale (CFS), and Trauma-Specific Frailty Index (TSFI)—in forecasting postoperative complications and functional outcomes in elderly patients with proximal humerus fractures (PHFs) treated either surgically or conservatively. *Materials and Methods:* A total of 244 patients aged ≥60 years with PHFs treated at Erzurum Hospital between January 2018 and January 2023 were included. Patients were categorized into surgical (n = 110) and conservative (n = 134) groups. Surgical procedures included open reduction and internal fixation (n = 88), hemiarthroplasty (n = 10), and reverse shoulder arthroplasty (n = 12). Frailty was retrospectively assessed using mFI-5, EFS, CFS, and TSFI based on 24-month follow-up data. Outcomes included complications, reoperations, rehospitalizations, and functional results measured by the American Shoulder and Elbow Surgeons (ASES) score. *Results:* The overall complication rate was 13.1%, with nonunion being the most common. Reoperation and rehospitalization rates were 10.6% and 20%, respectively. The mean ASES score was 71.3 ± 15.2, with 60% of patients achieving good or excellent outcomes. Frailty scores, particularly mFI-5 and EFS, were significantly higher in the conservatively treated group compared to the surgical group (*p* < 0.01). Across both treatment modalities, patients with higher frailty scores had significantly increased complication rates; however, this effect was more pronounced in the surgical group. Multivariate logistic regression revealed that mFI-5 significantly predicted complications, reoperations, and rehospitalizations (*p* < 0.001). EFS was associated with reoperation risk (*p* = 0.018), while CFS and TSFI were not significantly correlated with any of the outcomes. *Conclusions:* Among the evaluated indices, mFI-5 showed the strongest predictive accuracy for adverse outcomes in elderly PHF patients. Notably, the negative impact of frailty was more evident among surgically treated patients. Routine frailty assessment may facilitate better risk stratification and individualized treatment planning in this population.

## 1. Introduction

Proximal humerus fractures (PHFs) are among the most frequent osteoporotic fractures in the elderly, accounting for approximately 5–6% of all fractures in the general population and nearly 10% in individuals aged 60 years and older [1]. With increasing life expectancy, the incidence of these fractures is rising, primarily due to age-related declines in bone mineral density, neuromuscular coordination, and resistance to falls [2,3]. While fracture morphology and treatment strategy influence recovery, there is growing recognition that patient-level factors—particularly frailty—play a pivotal role in determining both clinical and functional outcomes in this population [4,5]. Despite the substantial clinical burden of PHFs, current risk stratification models rarely incorporate frailty, and there remains a critical gap in the literature regarding which frailty indices most accurately predict adverse outcomes in this context. This study aims to address that gap by comparatively evaluating the prognostic performance of four established frailty indices in older adults with PHF, thereby providing evidence-based guidance to support individualized, risk-informed decision-making in geriatric orthopedic care.

While fracture pattern and treatment modality influence recovery, the patient’s overall health status, especially frailty, plays a decisive role in functional outcomes following PHFs [6]. Treatment options range from conservative approaches to surgical interventions such as locked plate osteosynthesis, hemiarthroplasty, and reverse shoulder arthroplasty. However, in elderly patients, surgical interventions carry a higher risk of complications due to diminished physiological reserves, multiple comorbidities, and slower tissue healing. Common complications include screw migration, intra-articular screw penetration, infection, and nonunion, all of which can impair recovery and functional capacity [7,8,9].

Frailty, defined as a state of decreased physiological reserve and diminished resistance to stressors, is increasingly recognized as a critical factor in outcome prediction for elderly patients [10]. Though often associated with surgical outcomes, frailty also affects recovery in conservatively managed trauma cases. Several validated frailty indices have been developed to assess this vulnerability, including the 5-item Modified Frailty Index (mFI-5), Edmonton Frail Scale (EFS), Clinical Frailty Scale (CFS), and Trauma-Specific Frailty Index (TSFI) [11,12,13,14]. These indices encompass a range of domains such as comorbidity burden, cognitive status, functional independence, and social support, enabling a multidimensional evaluation of patient risk [15,16,17].

Despite their widespread use in orthogeriatrics, including hip fractures, spinal surgeries, and arthroplasties, the comparative effectiveness of these frailty indices in predicting complications and functional outcomes specifically after PHFs remains unclear. Moreover, few studies have assessed their applicability across both surgical and conservatively managed PHF patients. This lack of data limits the integration of frailty assessment into routine orthopedic planning for this fracture type.

Therefore, this study aims to address this gap by comparing four established frailty indices, mFI-5, EFS, CFS, and TSFI, in predicting complication rates, reoperations, rehospitalizations, and functional outcomes following PHF in patients aged 60 and above. Functional recovery was assessed using the American Shoulder and Elbow Surgeons (ASES) Shoulder Score, a validated measure for evaluating pain and shoulder function [18,19].

In addition, the American Society of Anesthesiologists (ASA) Physical Status Classification was used to assess perioperative risk among surgical patients. By analyzing its relationship with frailty indices, this study also seeks to explore the utility of ASA scoring in geriatric fracture risk stratification [20].

Ultimately, by systematically evaluating the predictive utility of multiple frailty indices, this study aims to support evidence-based treatment decisions and improve perioperative planning and postoperative care strategies in the elderly population with PHFs.

## 2. Materials and Methods

### 2.1. Ethical Approval

This retrospective study was approved by the Ethics Committee of Erzurum City Hospital (Approval No: 36029-13.02.2025) and conducted in accordance with the Declaration of Helsinki. As per institutional and national regulations for retrospective research, individual informed consent for study participation was not required. However, all patients provided informed consent for their clinical treatments, and data were anonymized prior to analysis.

### 2.2. Study Design and Patient Selection

This retrospective cohort study included 244 patients aged 60 years and older who were treated for PHFs at the Orthopedics and Traumatology Clinic of Erzurum Hospital between January 2018 and January 2023. A total of 252 patients were initially screened. Eight patients were excluded: five declined surgery despite indication and did not adhere to follow-up, while three were lost to follow-up due to transfer to other facilities or loss of contact. These exclusions ensured consistency in follow-up and data completeness.

### 2.3. Treatment Allocation

**Figure 1:**
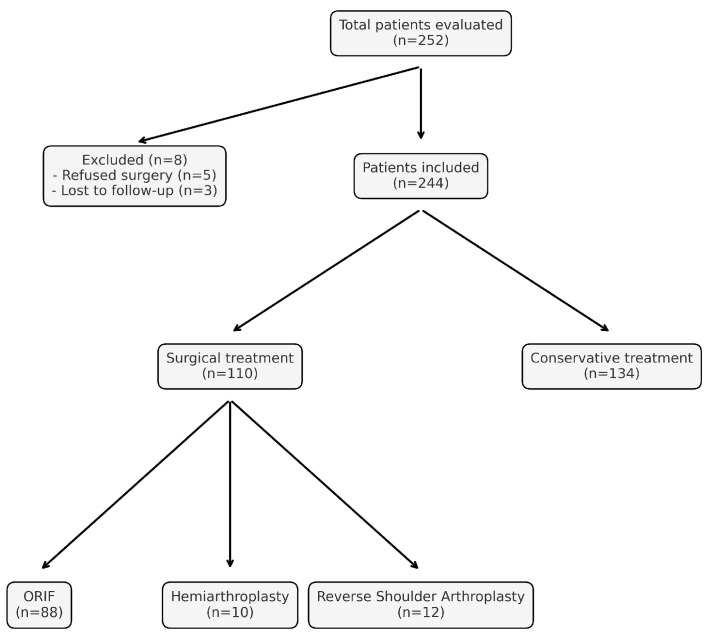
Patient selection and distribution by treatment modality.

A total of 110 patients underwent surgical treatment, and 134 were managed conservatively. Surgical procedures included open reduction and internal fixation (ORIF) with locking plates (n = 88), shoulder hemiarthroplasty (n = 10), and reverse shoulder arthroplasty (n = 12) (Figure 1).

Surgical intervention was performed in patients with displaced three- or four-part fractures (based on the Neer classification), preserved functional independence, and acceptable anesthesia risk (ASA I–II). Conservative management was preferred for non-displaced or minimally displaced fractures (e.g., Neer type I or II) and for patients with advanced age, high surgical risk, or limited functional demands.

### 2.4. Follow-Up Protocol

All patients were followed for a minimum of 24 months through structured outpatient visits at 6 weeks, 3 months, 6 months, 12 months, and 24 months. Functional outcomes, complications, radiographic healing, and frailty status were evaluated at each visit. Imaging included standard radiographs, and clinical assessments were standardized across both treatment groups.

### 2.5. Fracture Severity and Classification

Fracture morphology was assessed using plain radiographs and computed tomography (CT) and categorized according to the Neer classification system. This allowed the characterization of fracture complexity and guided treatment decisions. To ensure methodological consistency, fracture classification and treatment modality were used as covariates in the statistical analysis.

### 2.6. Inclusion and Exclusion Criteria

#### 2.6.1. Inclusion Criteria

1. Patients aged ≥ 60 years with unilateral PHF.
2. Diagnosis confirmed via physical exam, radiography, and CT.
3. First-time ipsilateral upper extremity fracture.
4. Minimum of 24 months follow-up.
5. Available and complete medical records.

#### 2.6.2. Exclusion Criteria

1. Multiple or bilateral fractures.
2. Pathological fractures.
3. History of previous shoulder or upper limb fractures.
4. Preoperative systemic infection or sepsis.
5. Neurological or malignant conditions causing pre-existing upper limb dysfunction.
6. Incomplete follow-up or missing clinical data.

### 2.7. Patient Demographics

The following demographic variables were recorded: age, gender, body mass index (BMI), smoking status, length of hospital stay, operation duration, re-hospitalization, re-operation, and complications. Complications were categorized as wound dehiscence or other non-surgical site infections, as well as pulmonary (e.g., embolism, pneumonia), cardiac (e.g., myocardial infarction, arrest), hematological (e.g., blood transfusion, deep vein thrombosis), renal (e.g., renal failure), nonunion, implant failure, osteonecrosis, and loss of reduction.

To classify life-threatening complications, the Clavien–Dindo classification system was used. Life-threatening conditions classified as Clavien–Dindo grade 4 included intensive care unit (ICU) admission, myocardial infarction, pulmonary embolism, postoperative dialysis requirement, re-intubation, and prolonged ventilation [21].

### 2.8. Frailty and Functional Assessment

All frailty indices were calculated using routinely collected clinical information, including admission notes, discharge summaries, physical therapy assessments, and standardized comorbidity documentation recorded in the hospital’s electronic health system. To minimize bias, scoring was performed by two independent evaluators who were blinded to postoperative outcomes and functional scores.

Both surgically and non-surgically treated patients were evaluated at the 24-month follow-up using four frailty indices: mFI-5, EFS, CFS, and TSFI.

For the mFI-5, variables such as diabetes mellitus, chronic obstructive pulmonary disease, congestive heart failure, hypertension, and functional dependence were identified. EFS scoring incorporated documented data on fall history, cognitive status, fatigue, and social support. The CFS was based on recorded levels of mobility and independence in daily activities. TSFI components, including nutritional status, activity level, and comorbidity burden, were derived from interdisciplinary clinical evaluations.

Shoulder functions were assessed using the ASES shoulder score. In addition, patients undergoing surgery were evaluated preoperatively with the ASA score to assess perioperative risk.

#### 2.8.1. ASA Score

Patients were preoperatively evaluated using the ASA classification to assess perioperative risk. The ASA score categorizes patients based on their overall health status. ASA 1 represents healthy individuals without systemic disease, demonstrating optimal health for surgery. ASA 2 includes patients with mild systemic diseases that do not limit daily activities and can tolerate surgery, such as those with hypertension or diabetes mellitus. ASA 3 comprises patients with severe systemic diseases that significantly impact general health, including conditions like coronary artery disease or severe asthma. ASA 4 involves patients with life-threatening systemic illnesses, such as multi-organ failure or severe heart failure. Finally, ASA 5 is assigned to moribund patients with minimal survival chance, often in terminal stages [22].

##### 2.8.2. mFI-5

The mFI-5 score is calculated based on five essential components: slow walking speed, weak grip strength, low physical activity level, weight loss, and fatigue. Patients are classified as having low frailty risk (0–1 points), moderate frailty risk (2–3 points), or high frailty risk (4–5 points). The mFI-5 is widely used to assess the frailty status of patients and predict postoperative outcomes [23].

##### 2.8.3. EFS

The EFS evaluates frailty through nine components: physical health, mental status, functional independence, social support, fatigue, pain, nutritional status, burden of illness, and history of falls. The cumulative score ranges from 0 to 18, with scores of 0–4 indicating a healthy status, 5–8 representing moderate frailty, and 9–18 indicating high frailty risk. The EFS is a comprehensive tool that integrates physical, cognitive, and social factors [24].

##### 2.8.4. CFS

The CFS rates patients from 1 to 9, reflecting their independence and overall frailty. CFS 1–4 denotes fit to vulnerable individuals with minimal frailty, CFS 5–6 indicates mild to moderate frailty, and CFS 7–9 reflects severe frailty or terminal illness. This scale is widely used to determine functional independence and predict recovery potential [25].

##### 2.8.5. TSFI

The TSFI incorporates various factors, including comorbid conditions, daily activity levels, health status, functional independence, and nutritional status. The score is calculated as the total score divided by 15, with values below 0.12 indicating non-frailty, 0.13–0.25 indicating pre-frailty, and values above 0.25 indicating frailty. This index is particularly useful for assessing trauma patients and predicting postoperative complications [26].

##### 2.8.6. ASES Shoulder Score

The ASES Shoulder Score is a widely accepted clinical tool used to evaluate shoulder function and pain. The score ranges from 0 to 100, with higher scores indicating better shoulder performance. A score of 90–100 reflects excellent function, 75–89 indicates good function, 50–74 shows moderate functional limitation, and 0–49 indicates poor function with severe limitations. The ASES score is a reliable outcome measure for assessing shoulder performance in both clinical and research settings [27]. The ASES score was recorded at the 24-month follow-up, following routine clinical evaluations at 6 and 12 months.

### 2.9. Statistical Analysis

Statistical analyses were performed using IBM SPSS Statistics for Windows, Version 26.0 (IBM Corp., Armonk, NY, USA). Continuous variables were presented as mean ± standard deviation (SD) or median (interquartile range [IQR]), based on the Shapiro–Wilk test for normality. Non-normally distributed variables were analyzed using non-parametric methods. Categorical variables were expressed as frequencies and percentages. Group comparisons were conducted using the independent *t*-test or Mann–Whitney U test for continuous variables, and the chi-square test or Fisher’s exact test for categorical variables. Correlations between frailty indices and ASES scores were assessed using Pearson’s or Spearman’s correlation coefficients. Multivariate logistic regression was used to identify predictors of complications, reoperation, and readmission. Multivariate logistic regression models were adjusted for age, sex, BMI, treatment modality, ASA class, and major comorbidities, including diabetes, hypertension, and coronary artery disease, to control for potential confounding. A *p*-value < 0.05 was considered statistically significant.

## 3. Results

### 3.1. Demographic Characteristics

This retrospective study included 244 patients with proximal humerus fractures. The mean age of the patients was 72.1 ± 8.2 years, with 103 (42.2%) being male and 141 (57.8%) being female. The average body mass index (BMI) was 27.5 ± 7.3 kg/m^2^, and 17.6% of the patients were identified as smokers.

The majority of patients (59%) were managed conservatively, while 41% underwent surgical interventions, including open reduction and internal fixation (ORIF), shoulder hemiarthroplasty, and reverse shoulder arthroplasty. The mean hospital stay duration was 3.3 ± 6.9 days.

Detailed demographic information of the patients is presented in Table 1.

### 3.2. Frailty Index Distribution

The mFI-5 demonstrated a mean score of 2.6 ± 1.7. Among the patients, 42% were classified as having low frailty (0–1), 38% as moderate frailty (2–3), and 20% as high frailty (4–5).

The EFS revealed a mean score of 7.8 ± 5.3, with most patients categorized within the mild to moderate frailty range.

The CFS yielded a mean score of 5.1 ± 2.7, indicating that moderate frailty was the most frequently observed category.

The TSFI showed a mean score of 0.51 ± 0.30, with the majority of patients falling within the prefrail to frail range (Table 2).

### 3.3. Relationship Between Frailty and Complications/Outcomes

Complications occurred in 13.1% of the cases. Among patients who developed complications, the most frequently observed type was nonunion, followed by pneumonia and pulmonary embolism (Table 3).

The comparative analysis of complication types revealed that the overall complication burden differed between treatment groups. While certain complications, such as osteonecrosis and pneumonia, were observed exclusively or more frequently in the surgical group, conservative management was associated with a lower incidence of major complications. Notably, the functional outcomes assessed using the ASES score were significantly more favorable in the conservative group (mean ASES: 75.0 vs. 66.8, *p* = 0.0001). Reoperation was required in 10.6% of cases, and 20% of patients were readmitted. All reoperations and hospital readmissions occurred exclusively in the surgical treatment group; no patients in the conservative group required conversion to surgery during the follow-up period.

Logistic regression analysis revealed that the mFI-5 score was significantly associated with an increased risk of complications (*p* < 0.001), reoperation (*p* < 0.001), and readmission (*p* < 0.001). In contrast, the Edmonton score was moderately associated with reoperation (*p* = 0.018) but not with complications or readmission. Neither the CFS nor the TSFI showed a significant association with any of the outcomes.

### 3.4. Visual Presentation

**Figure 2:**
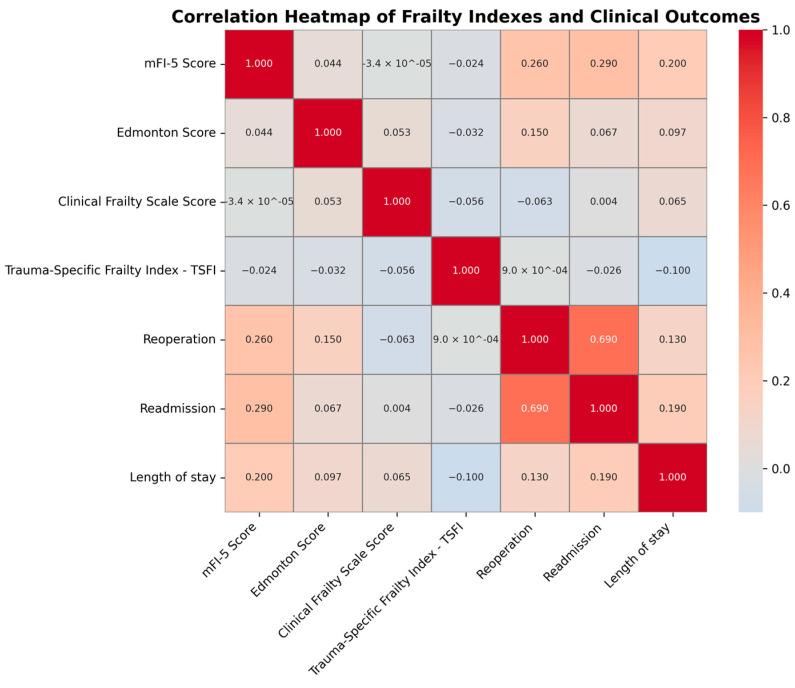
Correlation heatmap of frailty indices and clinical outcomes.

**Figure 3:**
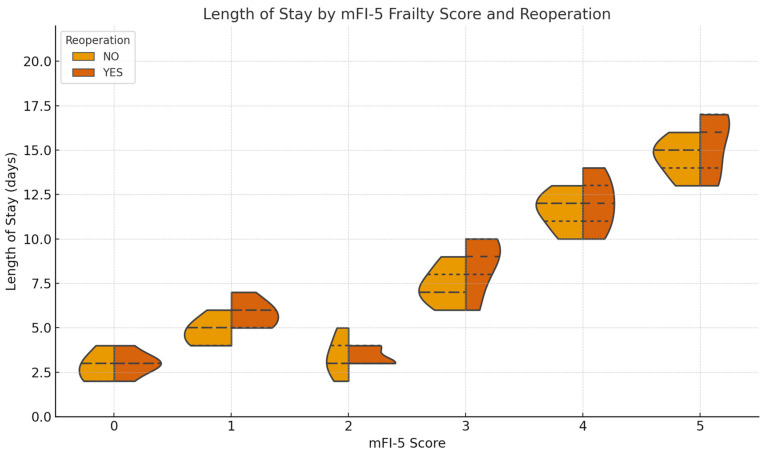
Distribution of length of stay according to mFI-5 Scores and reoperation status (Violin Plot). Note: The y-axis starts from zero to avoid misinterpretation. “Length of Stay” refers to the initial hospital admission following primary treatment.

**Figure 4:**
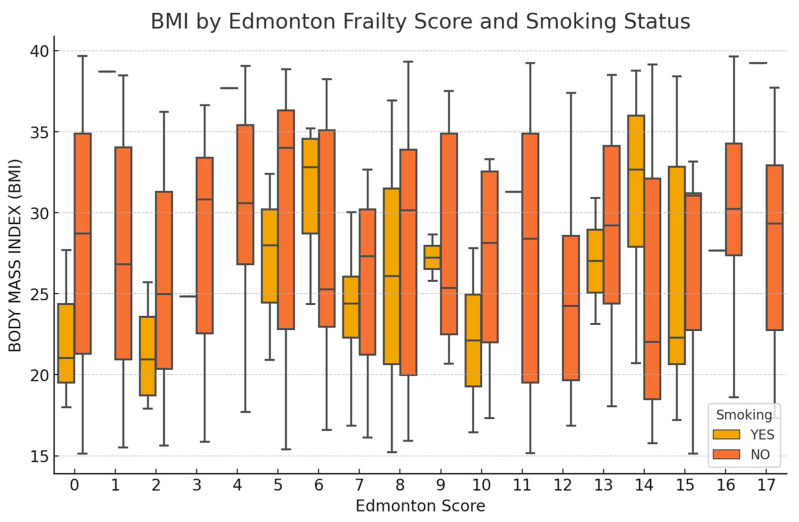
Distribution of Edmonton Score with BMI and smoking status (Boxplot).

**Figure 5:**
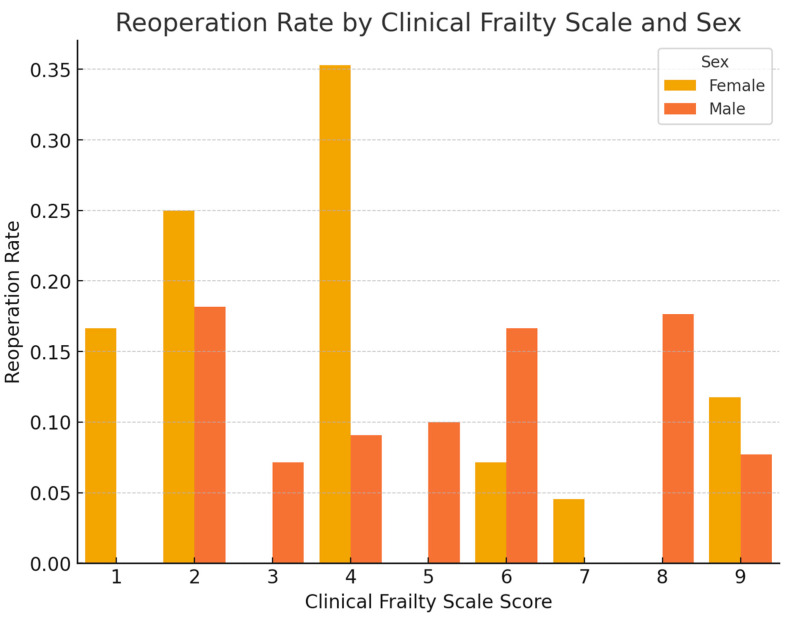
CFS and Reoperation Rate by gender. Bar graph demonstrating the relationship between Clinical Frailty Scale categories and reoperation rates, stratified by gender.

Figure 2, Figure 3, Figure 4 and Figure 5 comprehensively analyze the relationship between frailty index scores and key clinical outcomes. The correlation heatmap (Figure 2) illustrates the association between frailty indices (mFI-5, Edmonton, CFS, TSFI) and clinical outcomes (reoperation, readmission, length of stay).

The mFI-5 score shows a strong positive correlation with both reoperation and readmission, while the Edmonton score and CFS exhibit a weaker correlation with clinical outcomes. In contrast, TSFI shows no significant relationship with clinical outcomes.

High mFI-5 scores are associated with prolonged length of stay and increased reoperation rates (Figure 3).

In the Edmonton frailty score, a higher tendency for elevated BMI is observed at higher frailty levels. Smoking status is also associated with higher Edmonton scores (Figure 4).

CFS and Reoperation Rate (Bar Graph): Demonstrates that patients with higher CFS scores have an increased Reoperation Rate. This relationship is particularly pronounced in male patients (Figure 5).

TSFI and Readmission (Count Plot): Demonstrates that prefrail and frail patients have a higher readmission rate compared to non-frail patients.

**Figure 6:**
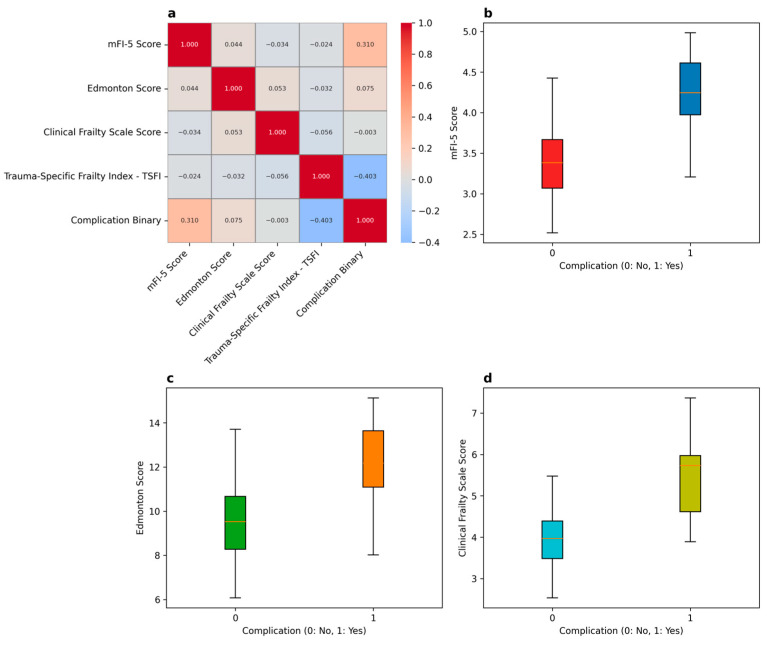
Overview of correlation between frailty scores and complications ((**a**) correlation heatmap, (**b**) mFI-5 vs. complications, (**c**) Edmonton vs. complications, (**d**) CFS vs. complications).

The visualizations show the correlation between frailty indices (mFI-5, Edmonton, Clinical Frailty Scale, TSFI) and complications. The mFI-5 score exhibits a strong positive correlation with complications, while the Edmonton score and Clinical Frailty Scale also demonstrate a positive correlation, though to a lesser extent. In contrast, TSFI does not display a significant correlation with complications (Figure 6a).

Higher mFI-5 scores are significantly associated with the presence of complications (Figure 6b). Elevated Edmonton scores correlate with an increased risk of complications (Figure 6c). Additionally, the Clinical Frailty Scale indicates that higher scores are linked to a greater risk of complications (Figure 6d).

Figure 6b–d clearly demonstrates that increasing frailty scores across different indices are consistently associated with elevated complication rates, with mFI-5 showing the strongest predictive value.

## 4. Discussion

This study evaluated the association between various frailty indices and treatment outcomes in elderly patients with PHFs. Our analysis identified mFI-5 as the most strongly associated index with complications, reoperation, and hospital readmission. This finding is consistent with previous literature emphasizing the clinical relevance of mFI-5 in orthopedic and surgical risk stratification. Segal et al. [28] demonstrated that mFI-5 is associated with 30-day postoperative complications in vertebral augmentation, while Panayi et al. [29] reported its correlation with complications, readmissions, and mortality across various surgical populations. These results highlight mFI-5 as a useful clinical tool for identifying high-risk patients, though it should be applied as part of a comprehensive assessment rather than as a stand-alone predictor.

PHFs are prevalent and complex injuries in elderly patients. Frailty has a critical impact on healing, complications, and overall functional outcomes. In this retrospective study, we examined four frailty indices—mFI-5, EFS, CFS, and TSFI—to assess their associations with clinical outcomes following both surgical and conservative treatments. Our findings support the use of frailty assessment in guiding individualized treatment planning.

The mFI-5 showed the strongest relationship with adverse outcomes. Patients with higher mFI-5 scores had more complications, prolonged recovery, and inferior functional results [30]. Fassiadis et al. [31] similarly described the utility of mFI-5 in forecasting postoperative complications in elderly individuals. These findings confirm its clinical relevance in perioperative risk evaluation.

Advanced age and comorbidities are well-established risk factors for adverse outcomes in elderly patients with PHFs. In line with recent studies, our data show that frail patients—particularly those undergoing surgery—experienced higher complication and readmission rates. Conversely, conservatively managed frail patients had more favorable trajectories. These findings highlight the interaction between biological frailty and treatment modality, reinforcing the need for individualized care plans. Our results align with the current literature emphasizing frailty-based risk stratification over chronological age alone [32].

The EFS was moderately associated with extended hospitalization and increased complication rates, particularly involving the cardiovascular and pulmonary systems. This may relate to its multidimensional structure, incorporating mental, nutritional, and systemic health assessments [33]. Previous research, including Elias et al. [34], supports its utility in perioperative planning.

The CFS demonstrated a weaker but still relevant association with treatment response and recovery duration. Its value may lie in its emphasis on general health status and patient independence, although subjectivity may limit consistency [35]. Lapner et al. [36] reported that CFS can help anticipate rehabilitation potential and treatment adherence.

The TSFI showed the least association with outcomes in this cohort. One likely reason is its original design for high-energy trauma populations. Many PHF cases, particularly conservatively managed ones, result from low-energy mechanisms, potentially reducing the sensitivity of TSFI in this setting [37]. Bhandari et al. [38] have also noted frailty as a factor in trauma recovery, but its clinical capture may vary depending on context.

In our cohort, patients undergoing surgery generally had higher frailty scores. Nevertheless, those with elevated frailty demonstrated worse outcomes postoperatively. Conversely, patients with similar frailty scores who were managed conservatively exhibited fewer complications and better functional outcomes. This suggests that frailty should influence the choice between operative and non-operative management, as also supported by prior studies [39,40].

Our findings contribute to a growing body of evidence that frailty status significantly impacts orthopedic outcomes. Ntritsos et al. [41] reported strong associations between frailty and complication rates, recovery timelines, and functional performance. Fassiadis et al. [31] further emphasized the utility of frailty indices for clinical planning and outcome prediction.

Importantly, these results underline the clinical importance of incorporating frailty assessment into orthopedic care. Frailty indices such as mFI-5, EFS, and CFS can aid in stratifying patient risk and tailoring interventions accordingly. However, these tools should complement—not replace—comprehensive clinical judgment.

This study has several limitations. Some are inherent to the observational and retrospective study design, including the reliance on past clinical records, which may introduce information bias or data entry inaccuracies. The lack of randomization precluded control over potential confounding variables. Moreover, this was a single-center study, which limits the generalizability of findings across broader healthcare settings with different patient populations or clinical practices. The inclusion of only patients with complete follow-up data may have led to selection bias, as those lost to follow-up were excluded.

In addition to these general methodological concerns, there are study-specific limitations that should be noted. Frailty indices were retrospectively scored using data from the 24-month follow-up, which—although based on clinically stable variables in older adults—does not fully replicate baseline prospective assessments. Furthermore, only the ASES score was used to evaluate functional recovery; other validated tools such as the Constant-Murley and DASH scores were not included, which may have limited the granularity of functional outcome assessment. Surgical technique varied between operating surgeons, which could have introduced procedural heterogeneity. Also, complications were recorded based on clinical documentation rather than standardized systematic screening, potentially leading to underreporting.

Importantly, no external validation was performed. Therefore, our findings, while internally consistent, require confirmation in future multicenter or prospective studies. Additionally, unmeasured variables such as socioeconomic status, baseline physical activity, or access to rehabilitation services may have influenced outcomes. While the sample size was sufficient for primary comparisons, some subgroup analyses may have been underpowered. Lastly, although a minimum follow-up of two years was ensured, long-term functional outcomes and quality-of-life metrics were not evaluated, limiting insight into sustained recovery.

These limitations should be carefully considered when interpreting the study’s conclusions and in designing future research to validate and extend our findings.

## 5. Conclusions

This retrospective cohort study demonstrated that preoperative frailty status is a significant determinant of clinical outcomes in elderly patients with proximal humerus fractures. Among the four evaluated indices, the Modified Frailty Index-5 (mFI-5) exhibited the highest predictive value for postoperative complications, reoperation, and hospital readmission. Patients with higher frailty scores were more vulnerable to adverse outcomes, particularly within the surgical cohort, whereas lower frailty scores correlated with more favorable results in conservatively managed patients. These findings emphasize the clinical relevance of frailty assessment and support its integration into individualized treatment planning. Future large-scale prospective studies are warranted to validate these associations and guide evidence-based orthopedic decision-making.

## Figures and Tables

**Table 1:** Distribution of demographic and frailty characteristics between surgical and conservative groups.

Variable	Category	Surgical (n)	Conservative (n)	*p*-Value
Age Group	65–74	49	55	0.6140
	75–84	33	35	0.6140
	85 and above	9	12	0.6140
	Under 65	19	32	0.6140
Sex	Female	65	76	0.8077
	Male	45	58	0.8077
Smoking	No	80	121	<0.001
	Yes	30	13	<0.001
ASA Classification	ASA I	9	0	1.0000
	ASA II	62	0	1.0000
	ASA III	36	0	1.0000
	ASA IV	3	0	1.0000
BMI Category	Normal weight	26	39	0.3049
	Obese	43	60	0.3049
	Overweight	21	19	0.3049
	Underweight	20	16	0.3049
mFI-5 Classification	High frailty	42	40	0.2750
	Low frailty	28	45	0.2750
	Moderate frailty	40	49	0.2750
EFS Classification	Mild frailty	11	19	0.4622
	Moderate frailty	17	14	0.4622
	No/minimal frailty	33	46	0.4622
	Severe frailty	49	55	0.4622
CFS Classification	Fit/Well	35	48	0.8268
	Mild frailty	22	28	0.8268
	Moderate-severe frailty	23	28	0.8268
	Very severe frailty	30	30	0.8268
TSFI Classification	Frail	86	100	0.1148
	Non-frail	16	30	0.1148
	Pre-frail	8	4	0.1148
ASES Functional Outcome	Excellent	17	36	0.0060
	Fair	42	33	0.0060
	Good	35	56	0.0060
	Poor	16	9	0.0060

Each variable is stratified into clinically meaningful categories, and group distributions are presented as frequencies (n). *p*-values are derived from chi-square tests. Statistically significant results (*p* < 0.05) are highlighted.

**Table 2:** Detailed demographic information according to the severity of frailty scores. The mFI-5 high frailty category was associated with a significantly increased risk of complications (OR: 3.45, 95% CI: 1.82–6.52, *p* < 0.001).

Frailty Index	Category	Age (Mean ± SD)	Male Count (%)	Female Count (%)	Smoking (%)	Length of Stay (Mean ± SD)	Reoperation Rate (%)	Readmission Rate (%)	Complication Rate (%)	*p*-Value
mFI-5	Low	71.8 ± 9.3	20 (55.6%)	16 (44.4%)	19.4%	1.9 ± 2.7	2.8%	11.1%	11.1%	0.007
	Moderate	71.9 ± 8.0	33 (37.1%)	56 (62.9%)	14.6%	3.5 ± 7.7	7.9%	13.5%	11.2%	0.023
	High	72.6 ± 8.5	31 (37.8%)	51 (62.2%)	24.4%	4.7 ± 8.3	22.0%	37.8%	34.1%	0.001
Edmonton	No/Mild	72.3 ± 8.2	21 (35.6%)	38 (64.4%)	11.9%	2.9 ± 6.1	3.4%	6.8%	10.2%	0.000
	Mild	74.3 ± 9.5	9 (30.0%)	21 (70.0%)	30.0%	2.3 ± 8.4	3.3%	16.7%	10.0%	0.085
	Moderate	70.4 ± 7.5	18 (58.1%)	13 (41.9%)	19.4%	3.2 ± 5.7	9.7%	32.3%	16.1%	0.408
	Severe	72.1 ± 8.4	44 (42.3%)	60 (57.7%)	17.3%	4.0 ± 7.7	16.3%	22.1%	21.2%	0.288
CFS	Fit	70.9 ± 8.0	37 (44.6%)	46 (55.4%)	18.1%	2.5 ± 4.6	10.8%	16.9%	14.5%	0.022
	Mild	73.2 ± 8.8	21 (42.0%)	29 (58.0%)	22.0%	3.3 ± 8.1	16.0%	28.0%	22.0%	
	Moderate	72.8 ± 7.8	15 (29.4%)	36 (70.6%)	13.7%	3.8 ± 8.3	5.9%	17.6%	11.8%	
	Severe	72.4 ± 8.4	30 (50.0%)	30 (50.0%)	16.7%	3.9 ± 7.4	10.0%	20.0%	21.7%	
TSFI	Non-frail	70.9 ± 8.1	20 (43.5%)	26 (56.5%)	21.7%	4.0 ± 9.5	8.7%	17.4%	15.2%	0.785
	Prefrail	69.7 ± 7.6	7 (50.0%)	7 (50.0%)	14.3%	5.8 ± 12.0	14.3%	28.6%	14.3%	
	Frail	72.6 ± 8.3	76 (41.3%)	108 (58.7%)	16.8%	2.9 ± 5.5	10.9%	20.1%	17.9%	

*p*-values indicate the statistical significance of the differences between groups. Kruskal–Wallis test was used for continuous variables without normal distribution, and the chi-square test was used for categorical variables.

**Table 3:** Comparison of specific postoperative complications and functional outcomes (ASES scores) between surgical and conservative treatment groups.

Complication Type	Conservative (n)	Surgical (n)	Surgical (%)	Conservative (%)	*p*-Value
Acute Kidney Failure	0	3	10.7%	0.0%	0.5390
Deep Vein Thrombosis	0	3	10.7%	0.0%	0.5390
Implant Failure	0	2	7.1%	0.0%	0.5447
Nonunion	2	6	21.4%	14.3%	0.6969
Osteonecrosis	0	3	10.7%	0.0%	0.5390
Pneumonia	1	4	14.3%	7.1%	0.6496
Pulmonary Embolism	1	3	10.7%	7.1%	1.0000
Wound Dehiscence	0	4	14.3%	0.0%	0.2829
ASES Score (Mean ± SD)	75.0 ± 17.2	66.8 ± 20.1			0.0001

*p*-values for individual complications were calculated using Fisher’s exact test due to small sample sizes. ASES scores were compared using the Mann–Whitney U test, and the results are presented as mean ± standard deviation. A *p*-value less than 0.05 was considered statistically significant.

## Data Availability

The raw data supporting the conclusions of this article will be made available by the authors upon request.

## Abbreviations

The following abbreviations are used in this manuscript:

mFI-5	Modified Frailty Index-5
EFS	Edmonton Frail Scale
CFS	Clinical Frailty Scale
TSFI	Trauma-Specific Frailty Index
PHFs	Proximal humerus fractures
ASA	American Society of Anesthesiologists
ASES	American Shoulder and Elbow Surgeons
ORIF	Open Reduction and Internal Fixation
CT	Computed Tomography
BMI	Body Mass Index
